# Organ culture storage of pre-prepared corneal donor material for Descemet's membrane endothelial keratoplasty

**DOI:** 10.1136/bjophthalmol-2016-308855

**Published:** 2016-08-19

**Authors:** Maninder Bhogal, Karl Matter, Maria S Balda, Bruce D Allan

**Affiliations:** 1 Department of Corneal and External Disease, Moorfields Eye Hospital, London, UK; 2 University College London, Institute of Ophthalmology, London, UK

**Keywords:** Cornea, Eye (Tissue) Banking, Imaging, Treatment Surgery, Wound healing

## Abstract

**Purpose:**

To evaluate the effect of media composition and storage method on pre-prepared Descemet's membrane endothelial keratoplasty (DMEK) grafts.

**Methods:**

50 corneas were used. Endothelial wound healing and proliferation in different media were assessed using a standard injury model. DMEK grafts were stored using three methods: peeling with free scroll storage; partial peeling with storage on the stroma and fluid bubble separation with storage on the stroma. Endothelial cell (EC) phenotype and the extent of endothelial overgrowth were examined. Global cell viability was assessed for storage methods that maintained a normal cell phenotype.

**Results:**

1 mm wounds healed within 4 days. Enhanced media did not increase EC proliferation but may have increased EC migration into the wounded area. Grafts that had been trephined showed evidence of EC overgrowth, whereas preservation of a physical barrier in the bubble group prevented this. In grafts stored in enhanced media or reapposed to the stroma after trephination, endothelial migration occurred sooner and cells underwent endothelial-mesenchymal transformation. Ongoing cell loss, with new patterns of cell death, was observed after returning grafts to storage. Grafts stored as free scrolls retained more viable ECs than grafts prepared with the fluid bubble method (74.2± 3% vs 60.3±6%, p=0.04 (n=8).

**Conclusion:**

Free scroll storage is superior to liquid bubble and partial peeling techniques. Free scrolls only showed overgrowth of ECs after 4 days in organ culture, indicating a viable time window for the clinical use of pre-prepared DMEK donor material using this method. Methods for tissue preparation and storage media developed for whole corneas should not be used in pre-prepared DMEK grafts without prior evaluation.

## Introduction

While new modalities show promise,^1^ at present, the only effective treatment for corneal endothelial dysfunction is transplantation. Isolated transplantation of the corneal endothelium with just its basement membrane, Descemet's membrane endothelial keratoplasty (DMEK), is a successful and increasingly popular treatment option. There is mounting evidence that DMEK may have inherent advantages over Descemet's stripping endothelial keratoplasty (DSEK) in terms of visual recovery,^2^ final acuity and rejection risk.^3^ ^4^ However, current evidence suggests that postoperative cell loss may be higher in DMEK than other forms of endothelial keratoplasty (EK) and this is likely to have a bearing on graft survival.^5–7^

While the number of DMEK procedures performed has increased over the past 5 years, it is yet to overtake DSEK, which remains the predominant form of EK in the USA.^8^ One of the reasons for the slow adoption of DMEK is fear of tissue wastage during preparation.^9^ Delivery of eye bank-prepared DMEK grafts might ease the learning curve and likely increase the adoption of DMEK, as it did for Descemet's stripping automated EK (DSAEK).^10^ Pre-preparation of graft tissue should therefore focus on minimising tissue wastage and ensuring that the transplanted tissue has the highest possible global endothelial cell density (ECD).

The endothelium of whole corneas has been well characterised after body temperature (31°C–37°C) storage—the system used widely across Europe. Changes in ECD during storage, endothelial wound healing,^11^ the spatial patterns of cell death and tight junction morphology have been evaluated.^12^ ^13^ This means that surgeons preparing DMEK grafts in theatre can be relatively assured of the quality and phenotype of the endothelial cells (ECs) being transplanted. However, little is known about how the corneal endothelium/Descemet's membrane (DM) complex behaves once separated from the stroma and returned to storage. We have shown that DMEK grafts carry over non-viable cells from the original period in culture, with further damage occurring from trephination and tissue handling.^14^ The second period of graft storage after DMEK preparation presents an opportunity to adjust media and storage conditions, enhancing wound healing and perhaps shortening the postoperative recovery period.

Strategies to improve endothelial wound healing and endothelial survival in whole corneas have included using serum,^15^ limiting storage time,^16^ changing media half way through the storage period and supplementing with peptide growth hormones known to be produced by the corneal keratocytes and epithelium.^17–20^ It is possible that grafts remaining attached to the stroma have improved viability and better wound-healing properties if a form of co-culture exists, and for those stored as free scrolls, enhancing the culture media with growth hormones may be beneficial, as it is for EC culture.^21^

We hypothesised that a serum and growth factor-supplemented organ culture media would be beneficial for preserving ECD and for promoting wound healing in DMEK grafts. The composition of this media was optimised in a porcine primary EC culture model in a manner similar to previously published work.^22^ In pilot experimentation, we observed a phenomenon of endothelial migration onto the stromal surface of DMEK grafts that were returned to culture after material preparation, a feature that, to our knowledge, has not been reported before. The degree of overgrowth and time period to first observation varied with the media and different storage methods and we systematically evaluated this in further experimentation.

We subsequently compared three contemporary methods for storing pre-prepared DMEK grafts. In method 1, the grafts are peeled off completely and shipped as a scroll in a glass phial containing organ culture fluid. This method is employed by the Amnitrans EyeBank in Rotterdam.^23^ In method 2, the grafts are peeled 90% off and then laid back on the stroma prior to transplantation.^24^ This is the predominant method followed in US eye banks working with cold storage media and has also been adopted by the Venice eye bank in an organ culture system. In method 3, also described by the Venice eye bank, the DM is separated from the stroma by creating a fluid bubble and then shipped with or without removing the stroma.^25^ ^26^

To our knowledge, no systematic comparison of global EC viability for these different storage techniques has been conducted, with most published studies reporting results of ECD changes in small high power fields.^26–28^ We previously described a technique to map the viability of every cell in DMEK transplants that fully account for graft curvature, wound healing, any proliferation and ongoing cell loss.^14^ We used this method to show that a peel technique was superior to a fluid bubble method in terms of endothelial viability for tissue prepared immediately prior to use. In this study, we evaluated all three storage methods to determine if there were any differences in cell phenotype or global viability after storage of pre-prepared grafts.

## Methods

**Figure 1:**
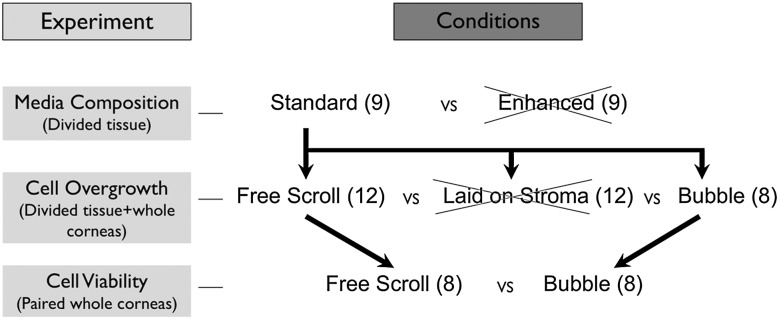
Flow diagram outlining the sequence of experimentation. The first experiment comparing storage media showed marked cell overgrowth with enhanced organ culture media making it unsuitable for use in Descemet's membrane endothelial keratoplasty (DMEK) storage. In the second experiment, DMEK grafts were returned to standard organ culture after preparation and were evaluated for features of abnormal cell overgrowth onto the stromal surface of the DM. Peeling and laying back on the stroma was associated with rapid cell overgrowth with endothelial-to-mesenchymal transformation and so was deemed unsuitable as a method for storing DMEK grafts. The other two preparation methods (bubble separation and storage as a free scroll) did not show overgrowth at 4 days post-preparation and global cell density assessment was performed using paired corneas.

A graphical representation of the sequence of experimentation is presented in figure 1. The study protocol was approved by the institutional review board at the Institute of Ophthalmology, University College London, Bath Street, London, UK. All procedures conformed to the tenets of the Declaration of Helsinki for biomedical research involving human tissues.

### Study tissue

Human corneoscleral buttons with consent for research use were obtained from Miracles in Sight (Winson Salem, North Carolina, USA) and UKTS Eye Banks (Bristol, UK). Tissue preparation and experimentation was performed by a single surgeon (Maninder Bhogal) with experience of >150 DMEK tissue preparations.

Tissue used for the wound healing and the initial endothelial overgrowth experiments had a minimum ECD of 1600 cells/mm^2^ and had been stored in organ culture for no longer than 5 weeks (media changed at week 4). Transplant grade tissue with EC counts of >2200 cells/mm^2^ and a storage time <14 days in Optisol GS (Bausch & Lomb Rochester, New York, USA) was used for global endothelial viability assessment and confirmatory experiments of EC overgrowth using whole corneas. In experiments comparing two variables, either the tissue was divided or paired tissue was used, with one whole cornea from each pair being assigned to each intervention, minimising the effects of donor variation.

Initial cell density measurement was performed in the eye bank supplying the tissue using either specular (Optisol) or bright field (organ-cultured tissue) microscopy. Each cornea was examined for gross cell death prior to experimentation by staining with 0.4% trypan blue (Sigma Aldridge Corporation, St Louis, Missouri, USA) for 60 s prior to viewing with an upright stereomicroscope. Samples were excluded from experimentation if they failed to meet the quality standards applied in our eye bank for transplant grade tissue; if there were large areas of cell loss; if there were >2% cell nuclei staining with trypan blue and if there was excessive tissue folding with cell death. Presence of guttae, central breaks in DM, positive donor serology and signs of media contamination also resulted in tissue exclusion.

### Media composition

Grafts were either stored in standard eye bank organ culture fluid (CorneaMax, Eurobio, Courtaboeuf, France) or stored in a growth factor-containing enhanced medium (En-OC). The formulation of En-OC was derived from preliminary experimentation in cultured porcine ECs (data not shown). The basal media was composed of equal quantities of minimal essential medium, Ham's F-12, M199 (PAA Laboratories, GE Healthcare Life Sciences, Pittsburgh, Pennsylvania, USA) and Optimem (Gibco, Thermo Fischer Scientific, Waltham, Massachusetts, USA) and supplemented with epidermal growth factor and basic fibroblast growth factor (10 ng/mL each, PeproTech, Rocky Hill, Connecticut, USA), insulin/transferrin/selenium mix 1:200 (Life Technologies), hydrocortisone (1 µg/mL), cholera toxin (100 ng/mL) and chondroitin sulfate (1 mg/mL) (Sigma Aldrich).

### Immunofluorescence

Markers indicative of a structurally normal endothelium (ZO-1), abnormal endothelial phenotype (α-smooth muscle actin (SMA)) and proliferating cells (Ki-67) were selected.

ZO-1 or α-SMA staining was performed with 1:300 diluted mouse antibody (Life Technologies, Carlsbad, California, USA). Ki-67 staining was performed using 1:400-dilution rabbit Ki-67 antibody (Sigma Aldrich). Actin staining was performed with 1:400 diluted AlexaFluor 488-conjugated phalloidin. Cy3-conjugated donkey anti-mouse IgG and AlexaFluor 488-conjugated goat anti-rabbit IgG (1:1000) were used as secondary antibodies (Life Technologies). Hoechst 33342 10 µM was used as a nuclear counterstain (Life Technologies).

The DMEK scrolls were removed from culture, washed in balanced salt solution (BSS) and fixed in 1% paraformaldehyde at room temperature for 5 min. Samples were permeabilised in Triton-X 1% for 5 min, blocked in bovine serum albumin for 30 min and incubated with primary antibody at 4°C for 24 hours. Samples were washed three times with phosphate-buffered saline and then incubated with secondary antibody and Hoechst 33258 overnight at 4°C. To assess specificity of the immunostaining, corneas were processed without primary antibody. DMEK specimens were flat-mounted in anti-fading mounting medium (Prolong Gold, Invitrogen) and whole mount images were acquired using the Nikon Eclipse Ti-E inverted fluorescence microscope (Nikon, Tokyo, Japan) and exported to ImageJ (National Institutes of Health, Bethesda, Maryland, USA) for analysis.

### Graft preparation techniques

We have previously described how we perform these techniques in detail.^14^

#### Free scroll preparation

Manual peeling was performed on the punch block of a standard trephine (Coronet, Network Medical, UK). After 360° peripheral scoring with a Sinsky hook, the DM/endothelium complex was peeled halfway, laid back flat and punched with an 8 mm trephine. The graft was then re-grasped, completely separated from the stroma and rinsed gently in BSS. Grafts were stored in a 24-well dish, lying on a glass cover slip to simulate storage in a glass phial.

#### Partially peeled with storage on the stroma

As with full peeling, peripheral scoring followed by partial peeling and trephination were performed. After trephination, the graft was re-grasped and peeled until only a small peripheral hinge of tissue remained (at least 90% of tissue peeled). The graft was then reapposed to the stroma using a ‘no-touch’ technique by wicking away fluid with a cellulose sponge. If sufficient fluid was removed from under the graft, it stayed apposed to the stroma after return to culture.

#### Fluid bubble separation

For fluid bubble separation, a 25-gauge needle was inserted underneath the trabecular meshwork (TM) and as close to DM as possible. Organ culture fluid was injected until a separation between the DM and stroma occurred (seen as a small peripheral ‘bubble’) and was continued until it extended to the TM in all areas. Once complete separation of the DM had been achieved, the needle was passed through the stroma and into the bubble space. Fluid was then withdrawn, allowing the bubble to collapse, reapposing DM to the corneal stroma. The corneas were placed endothelium up in a 6-well-dish containing 6 mL of organ culture fluid. The grafts were punched using an 8 mm trephine immediately prior to assessment.

### Determination of wound healing and proliferative capacity in DM separated from the stroma

**Figure 2:**
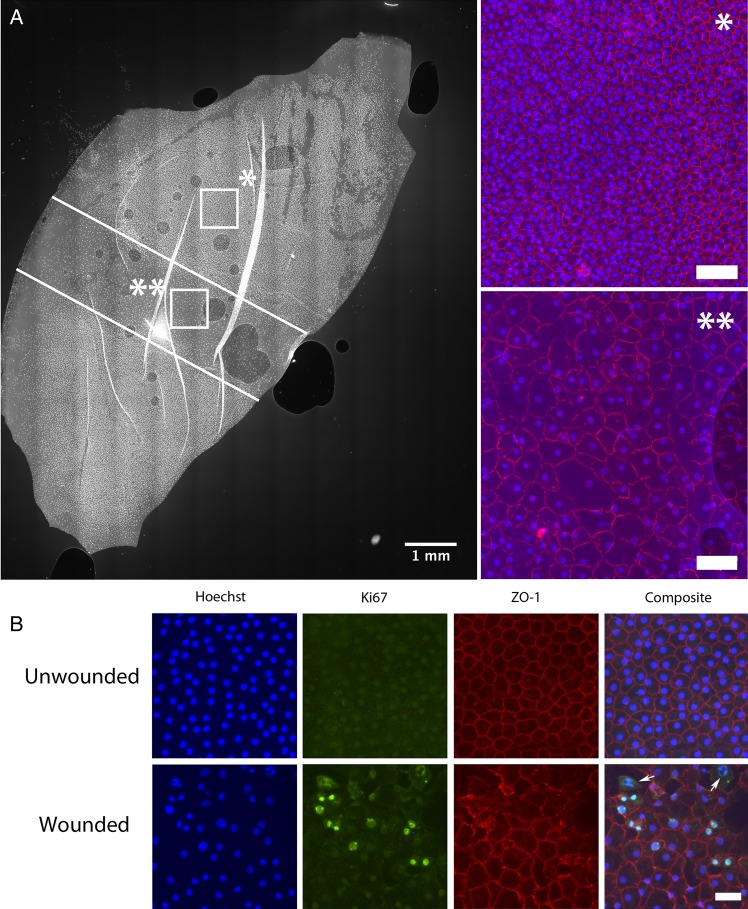
(A) Photomicrograph showing a Descemet's membrane endothelial keratoplasty (DMEK) graft from half a cornea. A standard injury is created with a silicon-tipped cannula (white lines). After 4 days in standard organ culture media, the wound has been completely covered by migrating endothelial cells. Cell density in the unwounded (*) area is much higher than in the wounded area (**). Tight junctions are not fully formed at 4 days in the wound area (ZO-1 in red). Scale bar 100 µm. (B) Half DMEK grafts were stained for makers of proliferation. Upper panel: Endothelial cells in unwounded areas of the half DMEK graft preserve a healthy, mature monolayer, confirmed by a normal staining for ZO-1. No Ki-67-positive (proliferating) cells are seen. Lower panel: Image taken from the wound site. Ki-67-positive nuclei (green) and cells in various stages of mitosis (white arrows) are seen. In spite of proliferation, cell density remains lower than in the unwounded areas. Scale bar 50 µm.

The corneal button was initially divided into two equal halves. A standardised 1 mm endothelial wound was produced across the centre of the cornea and perpendicular to the cut edge using a silicone-tipped cannula, similar to the method previously described in quartered corneas (figure 2A).^29^ Subsequently, DMEK scroll preparation was performed using the standard peel technique described earlier. Scrolled hemi-grafts were washed in BSS to remove any cellular debris, were placed in organ culture and were incubated at 37°C for variable periods ranging from 2 to 8 days. One half-scroll from each cornea was stored in standard culture media and the other half-scroll in En-OC, allowing for direct comparison of the effect of the different media on samples from the same donor. Samples were removed from culture at 2, 4, 6 and 8 days (n=3 at each time point) and were fixed. The area of DM not cover by cells was manually outlined and measured using ImageJ and divided by the starting wound area to calculate the percentage wound healing. Having determined the time necessary for full wound closure, three further corneas were analysed at day 4 (in total n=6). ECD and the percentage of Ki-67-positive (proliferating) cells were compared between wounded and unwounded areas and between the two different media.

### Assessment of EC overgrowth

Due to the limited availability of human corneal tissue, initial assessments were performed in divided samples (n=2 at each time point) for methods involving peeling. Corneal buttons were divided into quadrants using a scalpel. One quarter from each cornea was used for each of the first three groups. In the first group, DM was stripped completely and placed into standard culture media in a 48-well plate. In the second group, the same preparation procedure was performed but stripped DMs were placed in En-OC. In the third group, the DM was peeled 90% off in each quadrant and then laid back onto the stroma prior to storage. Experiments were repeated using six whole corneas for each group (n=2 at each time point). As bubble storage can only be performed with whole corneas, eight whole grafts were used to assess this method (n=2 at days 2 and 4, n=4 at day 8). Media was changed every 48 hours. Samples were removed and fixed at 2, 4 and 8 days and were stained with phalloidin and either ZO-1 or α-SMA. Each sample was carefully examined under the fluorescence confocal microscope for signs of endothelial migration onto the stromal surface. Samples were classified as positive for overgrowth if any whole cells could be seen migrating onto the stromal surface of the DM (cells were usually continuous with the endothelial monolayer and could be seen to wrap around the trephined edge of the graft).

### Global ECD assessment

Based on the findings of the cell overgrowth experimentation, grafts stored as free scrolls and after fluid bubble preparation were taken forward for global viability assessment (n=8 in each group). Grafts in both groups were stored for 4 days in standard organ culture fluid at 37°C prior to global viability assessment.

#### Fluorescence-based viability assessment

Each sample was covered with 250 μL of BSS containing Hoechst 33342 (10 μM), ethidium homodimer-1 (4 μM) and calcein-AM (2 μM) (Life Technologies) and was incubated at 37°C for 30 min prior to mounting on a customised curved viewing chamber. A Z-stack of 20–40 images was captured and in-focus information from each image was combined to enhance the depth of focus. Multiple image tiles were stitched together and individual cell viability assessment was performed across the entire graft surface using ImageJ (NIH, Bethesda, Maryland, USA). The total number of viable cells was divided by graft area (which had been corrected for imaging a curved surface) to calculate a global ECD. To control for differences in initial ECD, global ECD after storage was expressed as a percentage of eye bank-recorded specular central ECD (‘corrected global ECD’).

### Analysis

Images and numeric data were collated using a Filemaker Pro database (Apple, Cupertino, California, USA). Statistical comparisons were performed using Graphpad Prism (GraphPad Software, La Jolla, California, USA) using two-tailed t-tests with a threshold for statistical significance set at p<0.05.

## Results

### Wound healing assessment

The 1 mm wound had fully closed after 4 days in all corneas, in both standard organ culture and EN-OC (figure 2A). At day 2, the area of the wound not covered by the migrating cells was smaller in the En-OC group than in the standard organ culture group (39±7% vs 31±11%, n=3), but this failed to reach statistical significance. Evidence of EC proliferation was observed in most stored samples, but in general it was limited to the wound site and the areas of trephination damage (figure 2B).

Approximately 1% of all cells were Ki-67 positive with no difference in proliferative capacity observed with the use of En-OC. No Ki-67 positivity was seen in control samples fixed immediately after peeling without return to culture. Average ECD in the wounded areas was approximately half of the unwounded areas in both standard media (719±150 vs 1364±170 cells/mm^2^, p<0.001, n=8) and En-OC (863±166 vs 1540±209 cells/mm^2^, p<0.001, n=8). There was a trend towards higher ECD in the wounded areas of grafts stored in En-OC (863±166 vs 719±150, p=0.09, n=8) (see online supplementary table S1).

10.1136/bjophthalmol-2016-308855.supp1 Supplementary table

### Endothelial overgrowth

**Figure 3:**
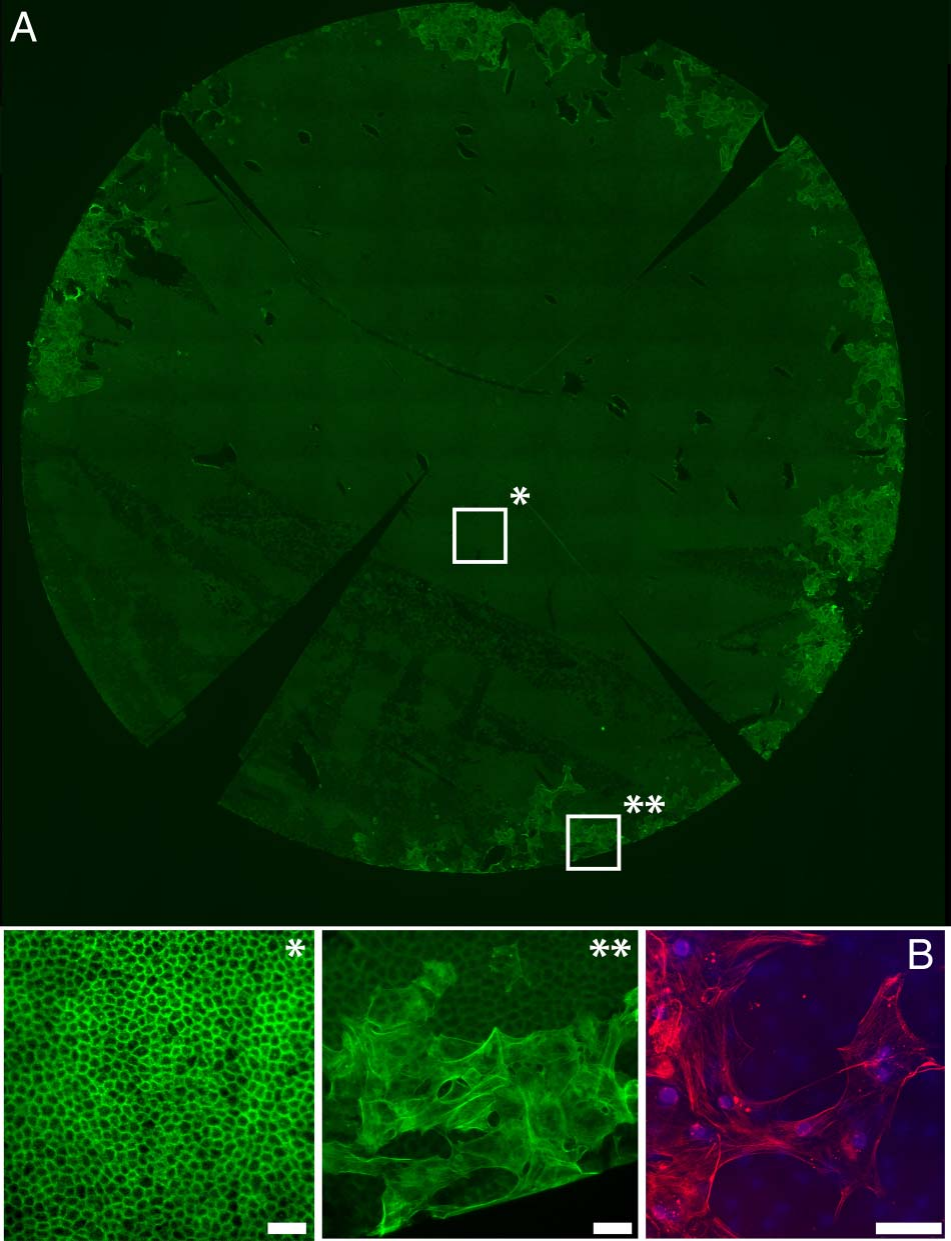
(A) Fluorescence image showing whole Descemet's membrane endothelial keratoplasty (DMEK) graft that has been peeled, laid back on to the stroma and stored for a further 4 days in standard organ culture media. The graft had been stained with phalloidin (green). *Enlarged segment from a central portion of the graft showing a healthy, hexagonal endothelial monolayer, with a normal actin staining pattern. **Enlarged segment from the periphery of the graft is shown. Large endothelial cells have migrated onto the stromal surface of the DM, with an out-of-focus layer of normal endothelial cells seen underlying these cells on the correct side of the DM. Cells have enlarged, flattened and acquired multiple linear stress fibres, indicating endothelial-to-mesenchymal transformation. (B) A confocal image of the migrating cells shows positive staining for α-SMA, a marker for endothelial-to-mesenchymal (red). Scale bars 50 µm.

The only storage method free from endothelial overgrowth was fluid bubble separation. No grafts stored as free scrolls in standard media had endothelial migration at day 4 but 50% showed this phenomenon at day 8. Endothelial overgrowth was more rapid and extensive in peeled tissue stored in growth factor-enhanced En-OC storage media, with migrating cells observed in all samples at day 2 and completely covering the stromal surface of graft by day 8. Overgrowth also occurred more quickly in grafts in which DM was incompletely peeled and laid back on the stroma with overgrowth seen in 50% of the samples by day 2 (see online supplementary table S2). Overgrowing cells in the samples stored laying on the stroma or incubated with growth factor-enhanced En-OC media lost junctional staining for ZO-1, had an increased number of actin stress fibres and stained positively for α-SMA (figure 3). Migrating ECs in the free scroll group retained a more ‘endothelial’ morphology with a rounded shape and fewer stress fibres.

10.1136/bjophthalmol-2016-308855.supp2 Supplementary table

### Global ECD

As excessive and early endothelial overgrowth was observed when DM grafts were partially peeled and laid back onto corneal stroma, this method was excluded from global endothelial viability assessment in transplant grade tissue. After 4 days in culture, global viable ECD was significantly higher in the free scroll donors than in the fluid bubble grafts (table 1).

**Table 1:** Graft characteristics and post-storage ‘corrected ECD’ in grafts stored as collapsed bubbles or free scrolls

	Free scroll OD (n=8)	Liquid bubble OS (n=8)	p Value
Donor age	65.4±10.3 years	65.4±10.3 years	
ECD±SD (specular)	2667±221 cells/mm^2^	2754±200 cells/mm^2^	
Death to storage time	6.2±4.1 hours	6.2±4.1 hours	
Cause of death	Trauma (4) Subarachnoid haemorrhage (1) Myocardial infarction (3)	Trauma (4) Subarachnoid haemorrhage (1) Myocardial infarction (3)	
Donors with diabetes	1 (in myocardial infarction group)	1 (in myocardial infarction group)	
Corrected graft area±SD	58.1±0.51 mm^2^	59.4±0.73 mm^2^	
Corrected global ECD±SD	74.2±3%	60.3±6%	0.035

Tissue was from paired donors. Global viability was calculated by counting all viable cells on the graft and dividing this value by the graft area (adjusted for imaging a spherical cap). This figure was presented as a percentage of the starting central ECD measured in the eye bank – corrected global ECD.

ECD, endothelial cell density; OD, oculus dexter; OS, oculus sinister.

### Qualitative assessment of cell death and endothelial morphology

**Figure 4:**
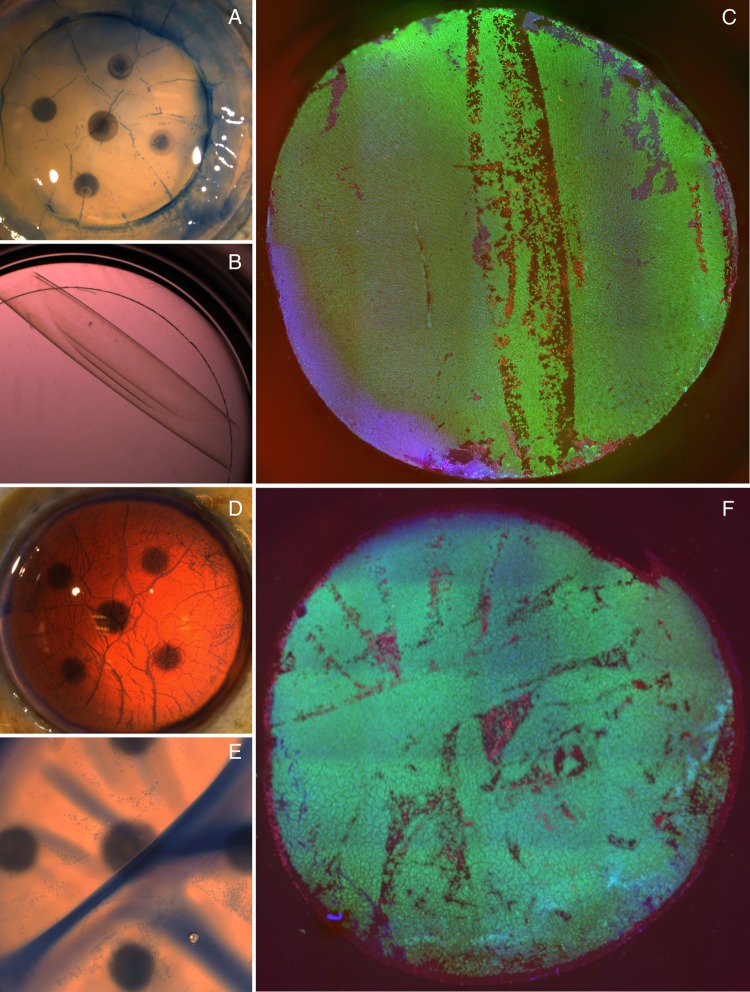
(A) A branching pattern of cell death, staining positively with trypan blue and corresponding to tissue folds, is seen immediately after preparation. (B) Scrolled graft is shown laying on a glass cover slip in a 48-well culture dish. (C) Global endothelial viability assessment showing characteristic lines of cell death corresponding to the long axis of the scroll. (D) Trypan blue staining immediately after bubble separation shows patterns of cell death consistent with tissue folds. (E) Trypan blue staining after tissue storage shows death cells at the peaks of tissue folds. (F) Global endothelial evaluation of bubble separated graft after trephination.

Characteristic and reproducible patterns of cell death were seen in both groups. In the free scroll group, linear patterns of cell death aligned with the long axis of the scroll were seen in all samples (figure 4A–C). In the bubble group, areas of cell death were frequently seen at peaks of tissue folds that had occurred as fluid reaccumulated between the DM and stroma (figure 4D–F).

Normal tight junction and actin staining patterns were observed in the healthy areas of graft from both groups, with no discernible differences between the two storage methods. Irregular staining was observed in areas of cell death and those adjacent to them.

## Discussion

As more surgeons adopt DMEK, there is an increasing interest in the delivery of pre-stripped DMEK donor tissue. The optimal storage method for pre-stripped grafts should maximise global graft viability, encourage areas of preparation damage to heal and ensure that the ECs are phenotypically normal.

A process of wound healing occurs in DMEK grafts that are returned to organ culture. A 1 mm wound (which is relatively large compared with typical hypotony-induced linear areas of cell death) heals in approximately 4 days. As with whole corneas, the process of wound healing occurs primarily by cell spreading with low-level mitosis limited to areas of wounding.^29^ ^30^ Cell density is significantly lower in the wounded area as compared with the surrounding unwounded areas showing that any proliferation is insufficient to restore the pre-wounding cell density.

Use of En-OC showed a trend towards increased cell density in the wounded area but it does not increase cell proliferation, as detected by Ki-67 staining, suggesting that the effect of growth factor supplementation is to primarily increase cell migration. The effect of growth factor supplementation in whole corneas is uncertain, with some studies showing minimal cell proliferation,^31^ while other reports suggest that significant levels of mitosis may occur.^29^ ^32^ Differences in donor age, cell density or pre-experimentation storage duration may account for the variations reported.^33^

In peeled and trephined grafts stored in the En-OC media, endothelial migration onto the stromal surface of DM occurred rapidly. While ECs on the anatomically correct side of DM maintained a normal phenotype (cortical actin staining pattern and ZO-1 expression at cell borders), cells migrating onto the stromal side lost ZO-1 expression, enlarged, flattened and had multiple pseudopodia. The cells had extensive actin stress fibres and stained positively for α-SMA. These features are suggestive of endothelial-mesenchymal transformation (EMT).^34^ Endothelial migration with a similar phenotype was seen in grafts peeled and laid back against the stroma, being observed as early as 2 days after return to culture. In free scrolls, migration was not observed in any grafts at day 4 post-preparation but was seen in grafts stored for longer periods to time, supporting the hypothesis that growth factors, either added to the media or released from the stroma, increased migration speed. In the bubble separation group, a physical barrier to migration is preserved, preventing overgrowth from occurring.

An EMT phenotype has been observed in some failed DMEK grafts and is associated with graft detachment and opacity.^35^ ^36^ Multi-layered, transformed ECs have also been observed in retrocorneal membranes in animal models of endothelial wounding.^37^ We were concerned to observe this phenomenon in DMEK donors stored in enriched media or in apposition to the stroma. It is unclear why cells migrating on the stromal surface of DM acquire a different phenotype to those migrating within healing wounds on the endothelial surface. Ichijima *et al*^38^ used two different terms for the behaviour of ECs following mechanical scraping and cryo-injury, cell spreading (collective cell movement with relative preservation of a normal EC phenotype) and cell migration (movement of mesenchymally transformed ECs), respectively.^39^ These processes are seen both in-vivo and ex-vivo^37^ ^40^ and, in other disease models, it can occur concurrently within the same microenvironment.^41^ In the samples we stored as peeled grafts laid back on the stroma or as free scrolls in enhanced organ culture media, cell *spreading* predominated in the wounded areas of the endothelial surface of the DM, whereas cell *migration* with loss of the normal phenotype predominated within the areas of overgrowth onto the stromal aspect of DM. In free scrolls, cell *spreading* predominated on both sides of DM over the time period examined.

It is possible that cell density and the time period to restoration of contact inhibition determine cell fate. On the endothelial surface of DM, in transplant grade tissue, cells re-establish contact reactively quickly and preserve normal borders on the non-migrating edge. On the stromal surface of DM, cells detach and migrate. Now unable to re-establish normal contact inhibition the cells produce numerous extensions and there is a loss of normal phenotype. These observations correspond with EC culture experiments showing a correlation between the fibrotic phenotype and low seeding density.^42^ We witnessed a density-dependent alteration in cell phenotype when one specimen with extensive cell loss was returned to storage (see online supplementary figure S1). In this specimen we observed an increasing derangement of the normal phenotype as cell density dropped, suggesting that cell density may also control cell fate in ex-vivo organ culture.

10.1136/bjophthalmol-2016-308855.supp3 Supplementary figure

EC migration off DM in divided whole corneas has been reported before,^33^ but EC migration onto the stromal side of DM has not been reported in DMEK donor tissue. Lie *et al*^27^ showed that repair of the trephination-induced damage area occurred in stored DMEK grafts; however, this was only examined using bright field microscopy, precluding any assessment of the relative contribution of cell division and spreading, and migration of ECs onto the stromal surface of the DM was not described.

As a period of 4 days is sufficient to prepare and distribute transplant tissue in most circumstances, we would recommend 4 days as a time limit for re-storage after scroll preparation for DMEK scrolls stored in standard organ culture conditions. Any alterations to media need careful testing specifically with DMEK tissue to define the safe time window for re-storage before new protocols are implemented clinically. Our enhanced En-OC media did not induce significant donor cell regeneration and does not seem beneficial for short-term storage of DMEK donors. Enhanced media may be useful if combined with the bubble separation method of DMEK preparation as a physical barrier to cell migration is preserved in this instance.

We previously found that a peeling technique is associated with less damage to the endothelium than bubble separation using our global corneal viability assessment method.^14^ However, a further reduction of approximately 20% in global ECD after storage was observed here in both groups, which is consistent with some other reports of DMEK storage.^26^ Storage of the DM in close proximity to the stroma failed to show any survival benefit, in keeping with the results of the grafts stored in enhanced media, suggesting that co-culture in proximity to other corneal cell types confers no survival benefit in the short term.

Areas of cell death conformed to characteristic patterns in both groups, with surrounding cells displaying changes in actin staining patterns. We suggest that a change in graft conformation induces a different pattern of mechanical stress, with new hotspots of cell death occurring at sites of maximal tension, similar to the mechanism thought to underlie cell death at corneal folds.^12^ In pilot experiments, we observed less cell death in corneas in which the partially peeled DM had been laid back on the stroma, thus maintaining a normal anatomical orientation. However, this storage method resulted in rapid endothelial migration and transformation, contaminating the stromal surface of DM. A method or device allowing maintenance of a normal DM curvature free from storage with the stroma may prevent the new patterns of cell loss we observed.

DMEK transplants stored in organ culture undergo a dynamic process of wound healing and ongoing cell death. Cell loss predominates and new patterns of cell death emerge as the mechanical forces placed on the ECs change. Methods for tissue preparation and storage developed for whole corneas should not be used in pre-prepared DMEK grafts without prior evaluation. Further work evaluating the storage of pre-prepared DMEK grafts in cold storage media is warranted as the process of EC healing and migration are known to be different than in organ culture.^43^

## References

[1] Okumura N, Koizumi N, Kay EP, et al The ROCK inhibitor eye drop accelerates corneal endothelium wound healing. Invest Ophthalmol Vis Sci 2013;54:2493–502. 10.1167/iovs.12-11320 23462749

[2] Guerra FP, Anshu A, Price MO, et al Descemet's membrane endothelial keratoplasty: prospective study of 1-year visual outcomes, graft survival, and endothelial cell loss. Ophthalmology 2011;118:2368–73. 10.1016/j.ophtha.2011.06.002 21872938

[3] Guerra FP, Anshu A, Price MO, et al Endothelial keratoplasty: fellow eyes comparison of Descemet stripping automated endothelial keratoplasty and Descemet membrane endothelial keratoplasty. Cornea 2011;30:1382–6. 10.1097/ICO.0b013e31821ddd25 21993468

[4] Anshu A, Price MO, Price FW Risk of corneal transplant rejection significantly reduced with Descemet's membrane endothelial keratoplasty. Ophthalmology 2012;119:536–40. 10.1016/j.ophtha.2011.09.019 22218143

[5] Monnereau C, Quilendrino R, Dapena I, et al Multicenter study of descemet membrane endothelial keratoplasty: first case series of 18 surgeons. JAMA Ophthalmol 2014;132:1192–8. 10.1001/jamaophthalmol.2014.1710 24993643

[6] Coster DJ, Lowe MT, Keane MC, et al A comparison of lamellar and penetrating keratoplasty outcomes: a registry study. Ophthalmology 2014;121:979–87. 10.1016/j.ophtha.2013.12.017 24491643

[7] Patel SV, Diehl NN, Hodge DO, et al Donor risk factors for graft failure in a 20-year study of penetrating keratoplasty. Arch Ophthalmol 2010;128:418–25. 10.1001/archophthalmol.2010.27 20385937 PMC3913733

[8] 2012 Eye Banking Statistical Report, Eye Bank Association of America. httpwww.restoresight.org

[9] van Zyl C, Terry MA DMEK: the Grand Prix ofcornea transplant surgery. Expert Review Ophthalmol 2014;9:89–98. 10.1586/17469899.2014.900440

[10] Price FW Jr, Price MO Evolution of endothelial keratoplasty. Cornea 2013;32(Suppl 1):S28–32. 10.1097/ICO.0b013e3182a0a307 24104929

[11] Doughman DJ Prolonged donor cornea preservation in organ culture: long-term clinical evaluation. Trans Am Ophthalmol Soc 1980;78:567–628. 7020215 PMC1312154

[12] Albon J, Tullo AB, Aktar S, et al Apoptosis in the endothelium of human corneas for transplantation. Invest Ophthalmol Vis Sci 2000;41:2887–93. 10967041

[13] Crewe JM, Armitage WJ Integrity of epithelium and endothelium in organ-cultured human corneas. Invest Ophthalmol Vis Sci 2001;42:1757–61. 11431439

[14] Bhogal M, Balda MS, Matter K, et al Global cell-by-cell evaluation of endothelial viability after two methods of graft preparation in Descemet membrane endothelial keratoplasty. Br J Ophthalmol 2016;100:572–8. 10.1136/bjophthalmol-2015-307534 26740609 PMC4819631

[15] Møller-Pedersen T, Hartmann U, Møller HJ, et al Evaluation of potential organ culture media for eye banking using human donor corneas. Br J Ophthalmol 2001;85:1075–9. 10.1136/bjo.85.9.1075 11520760 PMC1724125

[16] Redbrake C, Salla S, Frantz A Changes in human donor corneas preserved for longer than 4 weeks. Cornea 1998;17:62–5. 10.1097/00003226-199801000-00009 9436881

[17] Ehlers H, Ehlers N, Hjortdal JO Corneal transplantation with donor tissue kept in organ culture for 7 weeks. Acta Ophthalmol Scand 1999;77:277–8. 10.1034/j.1600-0420.1999.770306.x 10406145

[18] Imanishi J, Kamiyama K, Iguchi I, et al Growth factors: importance in wound healing and maintenance of transparency of the cornea. Prog Retin Eye Res 2000;19:113–29. 10.1016/S1350-9462(99)00007-5 10614683

[19] Lass JH, Musch DC, Gordon JF, et al Epidermal growth factor and insulin use in corneal preservation. Results of a multi-center trial. The Corneal Preservation Study Group. Ophthalmology 1994;101:352–9. 8115156 10.1016/s0161-6420(94)31329-7

[20] Barisani-Asenbauer T, Kaminski S, Schuster E, et al Impact of growth factors on morphometric corneal endothelial cell parameters and cell density in culture-preserved human corneas. Cornea 1997;16:537–40. 10.1097/00003226-199709000-00008 9294685

[21] Peh GS, Toh KP, Wu FY, et al Cultivation of human corneal endothelial cells isolated from paired donor corneas. PLoS ONE 2011;6:e28310 10.1371/journal.pone.0028310 22194824 PMC3241625

[22] Møller-Pedersen T, Hartmann U, Ehlers N, et al Evaluation of potential organ culture media for eye banking using a human corneal endothelial cell growth assay. Graefes Arch Clin Exp Ophthalmol 2001;239:778–82. 10.1007/s004170100354 11760040

[23] Ciechanowski PP, Droutsas K, Baydoun L, et al [Standardized Descemet membrane endothelial keratoplasty (DMEK): technique and latest results]. Ophthalmologe 2014;111:1041–9. 10.1007/s00347-013-3014-8 24763689

[24] Veldman PB, Dye PK, Holiman JD, et al Stamping an S on DMEK donor tissue to prevent upside-down grafts: laboratory validation and detailed preparation technique description. Cornea 2015;34:1175–8. 10.1097/ICO.0000000000000522 26147839

[25] Parekh M, Ruzza A, Salvalaio G, et al Descemet Membrane Endothelial Keratoplasty tissue preparation from donor corneas using a standardized submerged hydro-separation method. Am J Ophthalmol 2014;158:277–285.e1. 10.1016/j.ajo.2014.04.009 24792104

[26] Salvalaio G, Parekh M, Ruzza A, et al DMEK lenticule preparation from donor corneas using a novel “SubHyS” technique followed by anterior corneal dissection. Br J Ophthalmol 2014;98:1120–5. 10.1136/bjophthalmol-2013-304466 24879810

[27] Lie JT, Birbal R, Ham L, et al Donor tissue preparation for Descemet membrane endothelial keratoplasty. J Cataract Refract Surg 2008;34:1578–83. 10.1016/j.jcrs.2008.05.036 18721723

[28] Heindl LM, Riss S, Adler W, et al Corneal graft alterations after Descemet stripping: implications for split cornea transplantation. JAMA Ophthalmol 2013;131:687–9. 10.1001/jamaophthalmol.2013.794 23699848

[29] Senoo T, Joyce NC Cell cycle kinetics in corneal endothelium from old and young donors. Invest Ophthalmol Vis Sci 2000;41:660–7. 10711678

[30] Treffers WF Human corneal endothelial wound repair. In vitro and in vivo. Ophthalmology 1982;89:605–13. 10.1016/S0161-6420(82)34757-0 6181449

[31] Hoppenreijs VP, Pels E, Vrensen GF, et al Corneal endothelium and growth factors. Surv Ophthalmol 1996;41:155–64. 10.1016/S0039-6257(96)80005-1 8890441

[32] Slettedal JK, Lyberg T, Røger M, et al Regeneration with proliferation of the endothelium of cultured human donor corneas with extended postmortem time. Cornea 2008;27:212–19. 10.1097/ICO.0b013e31815b9723 18216579

[33] Patel SP, Bourne WM Corneal endothelial cell proliferation: a function of cell density. Invest Ophthalmol Vis Sci 2009;50:2742–6. 10.1167/iovs.08-3002 19218607 PMC2728347

[34] Roy O, Leclerc VB, Bourget J-M, et al Understanding the process of corneal endothelial morphological change in vitro. Invest Ophthalmol Vis Sci 2015;56:1228–37. 10.1167/iovs.14-16166 25698769

[35] Heindl LM, Schlötzer-Schrehardt U, Cursiefen C, et al Myofibroblast metaplasia after descemet membrane endothelial keratoplasty. Am J Ophthalmol 2011;151:1019–23.e2. 10.1016/j.ajo.2010.11.032 21457928

[36] Yum HR, Kim MS, Kim EC Retrocorneal membrane after Descemet membrane endothelial keratoplasty. Cornea 2013;32:1288–90. 10.1097/ICO.0b013e318296e0f7 23792439

[37] Petroll WM, Barry-Lane PA, Cavanagh HD, et al ZO-1 reorganization and myofibroblast transformation of corneal endothelial cells after freeze injury in the cat. Exp Eye Res 1997;64:257–67. 10.1006/exer.1996.0211 9176060

[38] Ichijima H, Petroll WM, Barry PA, et al Actin filament organization during endothelial wound healing in the rabbit cornea: comparison between transcorneal freeze and mechanical scrape injuries. Invest Ophthalmol Vis Sci 1993;34:2803–12. 8344802

[39] Friedl P, Alexander S Cancer invasion and the microenvironment: plasticity and reciprocity. Cell 2011;147:992–1009. 10.1016/j.cell.2011.11.016 22118458

[40] Petroll WM, Jester JV, Barry-Lane PA, et al Effects of basic FGF and TGF beta 1 on F-actin and ZO-1 organization during cat endothelial wound healing. Cornea 1996;15:525–32. 10.1097/00003226-199609000-00013 8862930

[41] McNiven MA Breaking away: matrix remodeling from the leading edge. Trends Cell Biol 2013;23:16–21. 10.1016/j.tcb.2012.08.009 22999190 PMC3905740

[42] Peh GS, Toh KP, Ang HP, et al Optimization of human corneal endothelial cell culture: density dependency of successful cultures in vitro. BMC Res Notes 2013;6:176 10.1186/1756-0500-6-176 23641909 PMC3659058

[43] Nejepinska J, Juklova K, Jirsova K Organ culture, but not hypothermic storage, facilitates the repair of the corneal endothelium following mechanical damage. Acta Ophthalmol (Copenh) 2010;88:413–19. 10.1111/j.1755-3768.2008.01490.x 19604163

