# Supplementary material for: Organ culture storage of pre-prepared corneal donor material for Descemet's membrane endothelial keratoplasty
Source: Br J Ophthalmol. 2016 Aug 19;100(11):1576–83. doi: 10.1136/bjophthalmol-2016-308855 (PMC5136687; doi:10.1136/bjophthalmol-2016-308855)
Supplement: Supplementary table [file bjophthalmol-2016-308855supp_table1.pdf]

	Media		
	Standard (n=8)	Enhanced (n=8)	
Donor Age	68.8±11.4		
Death to storage time	26.4±10.3		
Time in Organ Culture	33 days		
Cause of Death	Respiratory failure (2), Cerebrovascular accident (3), Malignancy (3)		
Donors with diabetes	2 (1CVA, 1 Respiratory failure)		
ECD prior to experimentation	1833± 155 cells/mm^2		
Unwounded ECD (cells/mm^2 ±STD)	1364 ± 170	1540 ± 209	p=0.19
Wounded ECD (cells/mm^2 ±STD)	719 ± 150	863 ± 166	p=0.09
	p<0.001	p<0.001	

Supplementary Table 1) Graft characteristics and endothelial cell of grafts stored in standard and enhanced culture media.
