# Supplementary material for: Organ culture storage of pre-prepared corneal donor material for Descemet's membrane endothelial keratoplasty
Source: Br J Ophthalmol. 2016 Aug 19;100(11):1576–83. doi: 10.1136/bjophthalmol-2016-308855 (PMC5136687; doi:10.1136/bjophthalmol-2016-308855)
Supplement: Supplementary table [file bjophthalmol-2016-308855supp_table2.pdf]

Condition	% of samples with Endothelial Overgrowth (N)			Comments
	Day 2	Day 4	Day 8	
Free Scroll (standard OC)	0% (4)	0% (4)	50% (4)	Few actin stress fibers, circular cells
Free Scroll (enhanced OC)	100% (4)	100% (4)	100% (4)	Flat cells with multiple extensions and excessive migration
Peel and lay on stroma (standard OC)	50% (4)	100% (4)	-	Flat cells with multiple extensions and excessive migration
Bubble (standard OC)	0% (2)	0% (2)	0% (4)	No cell overgrowth

Supplementary Table 2) Time point at which endothelial overgrowth was observed.

(N) refers to the number of samples in each group. OC = organ culture medium.
