# Supplementary material for: Organ culture storage of pre-prepared corneal donor material for Descemet's membrane endothelial keratoplasty
Source: Br J Ophthalmol. 2016 Aug 19;100(11):1576–83. doi: 10.1136/bjophthalmol-2016-308855 (PMC5136687; doi:10.1136/bjophthalmol-2016-308855)
Supplement: Supplementary figure [file bjophthalmol-2016-308855supp_figure.pdf]

Supplementary Figure

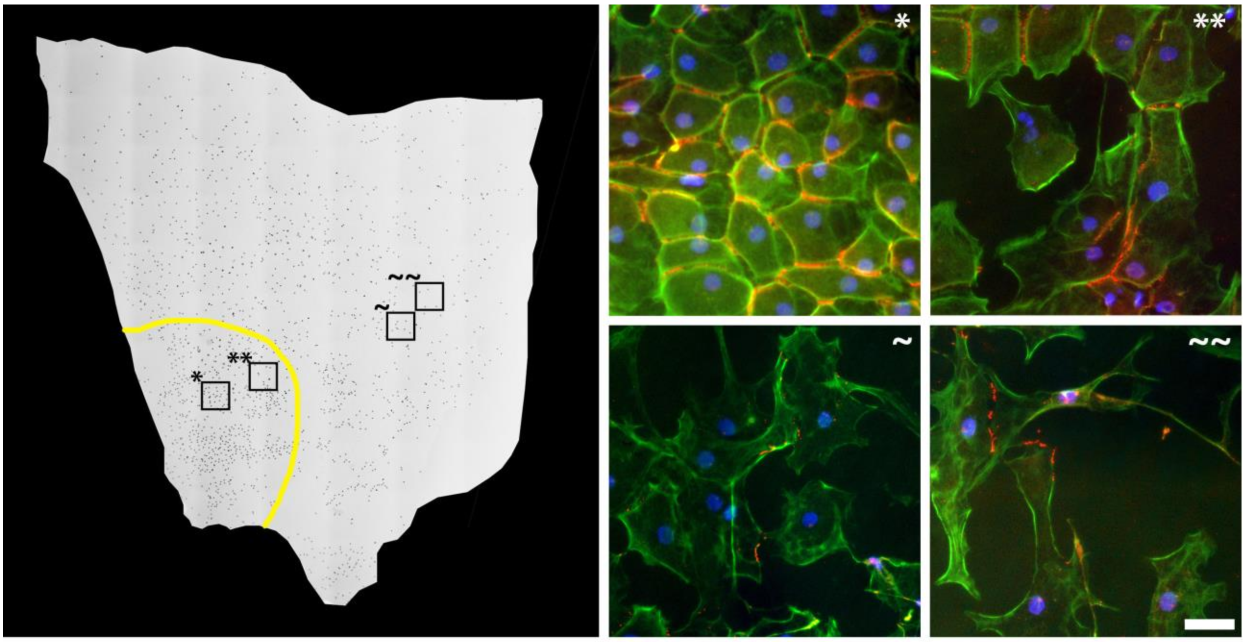

Supplementary Figure 1) Photomicrograph of a DMEK graft from one quarter of the cornea. Endothelial cells have been removed from all areas apart from one portion (yellow outline) using a silicone cannula. The graft was returned to culture in standard media for 8 days. Endothelial cells are seen to spread out from the healthy area. Where contact inhibition is restored and density maintained, a relatively normal phenotype is observed. As cells spread, density drops and they develop a more abnormal branching pattern with prominent actin stress fibers, similar to the pattern observed in cells migrating onto the stromal surface of DM.
